# Reviving Data Potential—The Reanalysis-Driven Metabolomics Data Repository MB-POST

**DOI:** 10.1021/acs.analchem.6c00697

**Published:** 2026-07-27

**Authors:** Yushi Takahashi, Akiyasu C. Yoshizawa, Takato Kiuchi, Ryosuke Hayasaka, Taihei Torigoe, Masatomo Takahashi, Takaki Oka, Yuki Matsuzawa, Motohiro Ogawa, Yoshihiro Izumi, Hiroshi Tsugawa, Akiyoshi Hirayama, Fumio Matsuda, Shujiro Okuda

**Affiliations:** † Medical AI Center, 12978 Niigata University School of Medicine, 2-5274, Gakkocho-dori, Chuo-ku, Niigata 951-8514, Japan; ‡ Institute for Advanced Biosciences, 74060 Keio University, Kakuganji 246-2, Mizukami, Tsuruoka-shi, Yamagata 997-0052, Japan; § Mass Spectrometry Center, Graduate School of Science, 12923 The University of Osaka, 1-1 Machikaneyama, Toyonaka, Osaka 560-0043, Japan; ∥ Department of Biotechnology and Life Science, 13125 Tokyo University of Agriculture and Technology, 2-24-16 Naka-Cho, Koganei-shi, Tokyo 184-8588, Japan; ⊥ Graduate School of Pharmaceutical Sciences, Kyoto University, 46-29 Yoshida-Shimoadachi-cho, Sakyo-ku, Kyoto 606-8501, Japan; # Department of Bioinformatic Engineering, Graduate School of Information Science and Technology, 13013 The University of Osaka, 1-5, Yamadaoka, Suita, Osaka 565-0871, Japan

## Abstract

Metabolomics investigates an extraordinarily broad chemical space and generates experimental data with substantial potential for reuse in chemistry, biology, and related fields. We developed MB-POST (https://repository.massbank.jp), a new metabolomics mass spectrometry data repository based on a concept distinct from that of existing platforms, to fully realize this potential. MB-POST implements a reanalysis-oriented metadata framework that captures the information essential for data reuse while providing a streamlined submission system that enables the rapid deposition of standardized metabolomics metadata together with experimental data. Since its public pilot release in December 2024, MB-POST has attracted strong community adoption, with more than 170 experimental projects deposited within its first year and a half. Thus, MB-POST provides a practical foundation for the large-scale reuse of metabolomics mass spectrometry data and represents a concrete step toward accelerating open and data-driven scientific research.

## Graphical abstract

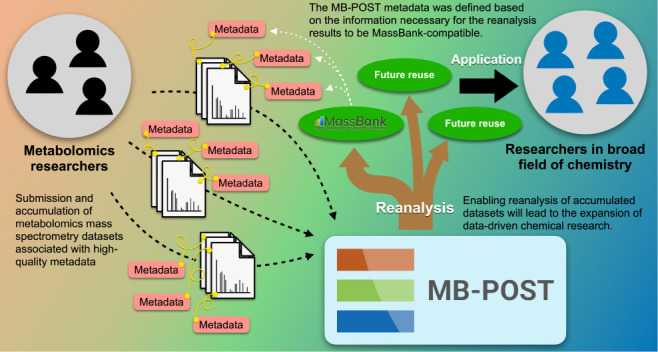

## Introduction

We developed a metabolomics mass spectrometry data repository, MB-POST, that enables rapid data upload with minimal barriers to data submission to provide a robust data source for information-driven research across chemistry and related disciplines. More than 170 independent projects were deposited within one year and a half of the pilot release. In this paper, we describe the design and implementation of the repository.

Data and computations now serve as the central engines of discovery, extending the scope of chemistry beyond conventional boundaries. This transformation critically depends on the availability of large reusable data sets. In chemistry, resources such as PubChem,^1^ complemented by specialized databases including KEGG COMPOUND^2^ (and its predecessor KEGG LIGAND^3^) and MassBank^4^ for compound identification, have long supported data-centric research.

Metabolomics spans a vast scientific field, from biochemistry to environmental chemistry, and systematically generates comprehensive molecular data. Since the 2009 Toronto Agreement,^5^ data deposition through public repositories has become effectively required for omics research. These data sets are intended for reuse, and from an open-science perspective, particularly under Findable, Accessible, Interoperable, and Reusable (FAIR) data principles,^6^ the public availability of the data itself constitutes a recognized scientific contribution, even in the absence of accompanying analyses.

First, we would like to discuss what types and quantities of data should be accumulated, as well as how such data should be made publicly available. Genomic and transcriptomic data are centralized in a relatively small number of data repositories. However, this model is impractical in proteomics because of the large size of raw mass spectrometry data. Instead, multiple data repositories in the field of proteomics coexist while forming an interconnected network under the ProteomeXchange^7^ consortium, which facilitates collaboration among them (see Table S1 in the Supporting Information for a comparison of metadata item number across existing repositories (MetaboLights,^8^ Metabolomics Workbench,^9^ MassIVE^10^/GNPS,^11^ MetaboBank,^12^ and KMAP^13^) and MB-POST in this study). Given the methodological and technical similarities between proteomics and mass spectrometry-based metabolomics, a comparable federated ecosystem is highly likely to emerge in metabolomics. The development of new data repositories is unlikely to be accepted in fields such as genomics, where data storage is centralized in a small number of repositories. In contrast, however, it is desirable to deploy multiple repositories in fields where a networked data repository ecosystem is operational, from both disk capacity and scalability perspectives.

Furthermore, accurate metadata describing experimental and analytical conditions are essential for meaningful data reuse, particularly for computational reanalysis. In omics research, public data repositories generally adhere to domain-specific minimum information standards that define essential metadata required for data deposition and reuse. These community-endorsed standards play a central role in ensuring data interoperability, reproducibility, and long-term usability. In contrast, however, a universally accepted minimum information standard for metabolomics has not yet been established. Consequently, the scope and structure of the collected metadata vary substantially across metabolomics data repositories (Table S1). Each repository uses its own information format, such as Investigation/Study/Assay (ISA)-Tab,^14^ mwTab,^15^ and ReDU.^16^

The primary reason for this is that, in the absence of established minimum information standards, there is a tendency to require more metadata fields to be filled out in anticipation of the development of new analytical methods in the future. However, this is likely to raise the barrier for depositing data for the submitters. In addition, while manual curation may be performed at the time of submission to improve the quality and accuracy of metadata, this is also likely to impose a burden on submitters who are not specialists in metabolomics. Although inaccurate metadata must be avoided, an overly complex submission workflow risks discouraging data sharing and ultimately limits the accumulation of reusable data sets.

In proteomics, which is the omics field most closely aligned with metabolomics in terms of research methods, metadata compliant with MAGE-TAB for Proteomics^17^—based on the Minimum Information for Proteomics Experiment (MIAPE) standard^18^—is being collected. MAGE-TAB for Proteomics itself originated from MAGE-TAB,^19^ a framework originally established based on the Minimum Information About a Microarray Experiment (MIAME) standard.^20^ This enables data reuse, represented by data reanalysis in proteomics. In addition, for the Japan Proteome Standard Repository/Database (jPOST)^21^ for proteomics mass spectrometry data that we have developed, we have optimized the design of its user interface, eliminating the need for manual metadata curation during data submission.

Based on these considerations, we established the following three guiding principles for developing a new metabolomics data repository.

**Reanalysis-oriented practical metadata principle: Metadata focused on practical reanalysis.** Metadata items were selected by prioritizing the analytical workflows currently most widely used, enabling the systematic accumulation of reanalyzable data while minimizing unnecessary burden on users. Simultaneously, we carefully ensured that the selected items would be suitable for data reuse applications that require annotations of an uncompromising quality.
**Interrepository collaboration principle: Readiness for interrepository collaboration.** This system was designed to support future federation and interoperability among metabolomics repositories, analogous to existing proteomics networks. Anticipating the possibility that metadata standardization may be undertaken with the aim of establishing minimum information guidelines, our repository has been designed so that its metadata can be migrated to the standard metadata format once such a standardization is implemented.
**Proven infrastructure reuse principle: Reuse of proven infrastructure and user experience.** The repository inherits both the technical framework and familiar look and feel of our established platforms for mass-spectrometry-based omics data, including the jPOST repository for proteomics and GlycoPOST^22^ for glycomics. This continuity provided an intuitive user interface that enabled an efficient data transfer. Since proteomics analytical methods are most closely related to metabolomics, we made extensive use of the findings from the jPOST repository in the development process.

On the basis of these guiding principles, we developed the metabolomics data repository MB-POST, reported in this paper.

## Methods

### Principles Guiding the Compilation of Metadata

The effective reuse of mass spectrometry-based metabolomics data almost always requires reanalysis of the original data sets. This has become the norm in proteomics. From this perspective, our reanalysis-oriented practical metadata principle presented at the end of the previous section, that metadata should contain sufficient information to enable reanalysis, is both reasonable and necessary.

This principle is grounded in practical experience. Our group continues to reanalyze public metabolomics data sets in MB-POST, evaluate the resulting spectra, and incorporate validated entries into MassBank, a curated reference library for metabolite mass spectra (manuscript in preparation). Because MassBank registration requires high confidence and reproducibility, we defined a successful reanalysis leading to the inclusion of reference spectra as a concrete target use case.

The second guiding principle, the interrepository collaboration principle, was interoperability among repositories. Recent activity of the coordination framework MetabolomeXchange^23^ has been limited. In parallel, a new international initiative called the MetabolomicsHub consortium,^24^ supported by Chan Zuckerberg Biohub and including key contributors from the Proteomics Identifications Database (PRIDE),^25^ began coordinating metabolomics repositories, with our group participating as one of the stakeholders. This suggests that metabolomics successfully follows the ProteomeXchange^7^ model.

Therefore, we designed our metadata schema to be compatible with future harmonization efforts. Specifically, while drawing extensively on the well-established metadata practices of proteomics, we ensured that our framework can be readily adapted or replaced should a community-endorsed minimum information standard for metabolomics be formally defined by an international group.

Specifically, we adopted the metadata schema of the *Journal of Proteome Data and Methods* (JPDM), which extends jPOST metadata with sample and data relationship format (SDRF)-equivalent information from MAGE-TAB for Proteomics, demonstrating large-scale reanalysis. Metadata elements from MetaboLights, Metabolomics Workbench, and MetaboBank were then mapped to this schema. Each item was evaluated through consensus among metabolomics researchers and curated solely on the basis of its necessity for (re)­analysis (see Table S2 for details).

### System Implementation and Robust File Uploads

The MB-POST system implementation builds upon the established architecture of jPOST/GlycoPOST in accordance with our third guiding principle, the proven infrastructure reuse principle. The user interface of MB-POST was developed using the React library (https://react.dev). MB-POST uses the PRESTO system^26^ (https://prestotools.github.io) for robust, efficient file uploads. The PRESTO system is specifically designed to enable the reliable transfer of extremely large raw data files generated by mass spectrometers by decomposing each data file into small “chunks” and uploading them to the repository server in parallel. This architecture substantially improves both the speed and error resilience of file uploads over the Internet.

In general, data transmission between geographically distant locations often suffers from severe degradation in transfer rate due to network latency.^26^ Chunk-based data upload effectively mitigates this issue. Moreover, even if transient failures occur in the network or on the server during an upload session, the entire upload process is not aborted. Instead, only the affected chunk files need to be retransmitted, enabling the upload to resume seamlessly and ensuring continuity of the overall process.

The PRESTO system requires only a standard web browser for operation and does not depend on any external software, such as file transfer protocol (FTP) client applications. As a result, users can complete the entire data submission workflow to MB-POST within their web browser, significantly lowering the technical barriers to data deposition. Figure S1 illustrates the conceptual framework for file uploading to MB-POST using the PRESTO system.

### Metadata Management

In conventional metabolomics repositories, experimental metadata are often submitted as spreadsheets. However, assigning appropriate metadata to tens or hundreds of data files generated from a single experiment is highly labor-intensive, placing a substantial burden on data submitters. In addition, free-text entry enables inconsistent and arbitrary descriptions, frequently resulting in heterogeneous metadata quality and the need for extensive manual curation.

MB-POST uses a dedicated web-based interface with a reusable metadata unit termed a “preset” to overcome these limitations. In MB-POST, a preset is a user-defined, reusable collection of semantically coherent metadata (e.g., sample information, instrument configuration, and analytical conditions) that can be shared across multiple data files. This enables users to assign identical metadata to multiple files simultaneously, eliminating redundant data entries while maintaining consistency. Users can create new presets *de novo* or generate them by partially modifying previously created presets, thereby enabling rapid and consistent metadata preparation for newly submitted data files. The MB-POST system organizes and manages user-defined presets in four predefined categories: “Sample”, “Preparation”, “Analytical condition”, and “Software setting”. Users could freely assign one or more presets from each category for each raw data file and flexibly combine them to define a preset profile that collectively describes the experimental context of the file.

A “Sample” preset is designed to describe intrinsic attributes of the analyzed sample itself. Users specify the sample category (e.g., biological or environmental sample) and information such as the organism, tissue, disease state (if applicable), and genotype for biological samples. In addition, users can specify whether each raw data file comes from the disease group or the control group. When relevant, an associated BioSample^27^ identifier is provided. A “Preparation” preset captures details of sample preparation procedures. This includes information on the fractionation methods, derivatization strategies, and use of internal standards. An “Analytical condition” preset describes the processes used for separation and detection of analytes, particularly those involving chromatographic systems and mass spectrometers. Users can specify method types (such as liquid chromatography–mass spectrometry (LC–MS), gas chromatography–mass spectrometry (GC–MS)) and instrument modes (such as data-dependent acquisition (DDA) high-resolution, DDA low-resolution, and data-independent acquisition (DIA)) using this preset, enabling MB-POST to accept a wide range of data, including both untargeted and targeted metabolomics data. A “Software setting” preset documents the software tools used to annotate the experimental results, along with the specific parameter settings applied during data analysis.

An exception is the replicate information for the samples, which must be specified on a per-file basis. Accordingly, this information should be provided via the upload of a dedicated Excel file rather than through the use of presets. A screenshot of the preset selection interface in MB-POST is shown in Figure S2.

For individual metadata fields such as organisms, mass spectrometer models, and ionization methods, the system presents frequently used terms as predefined options in drop-down menus. Terms not included in these lists can be efficiently selected by using an incremental search, which dynamically narrows the candidate terms based on partial inputs. An example of a preset input interface is listed in Figure 1.

**Figure 1:**
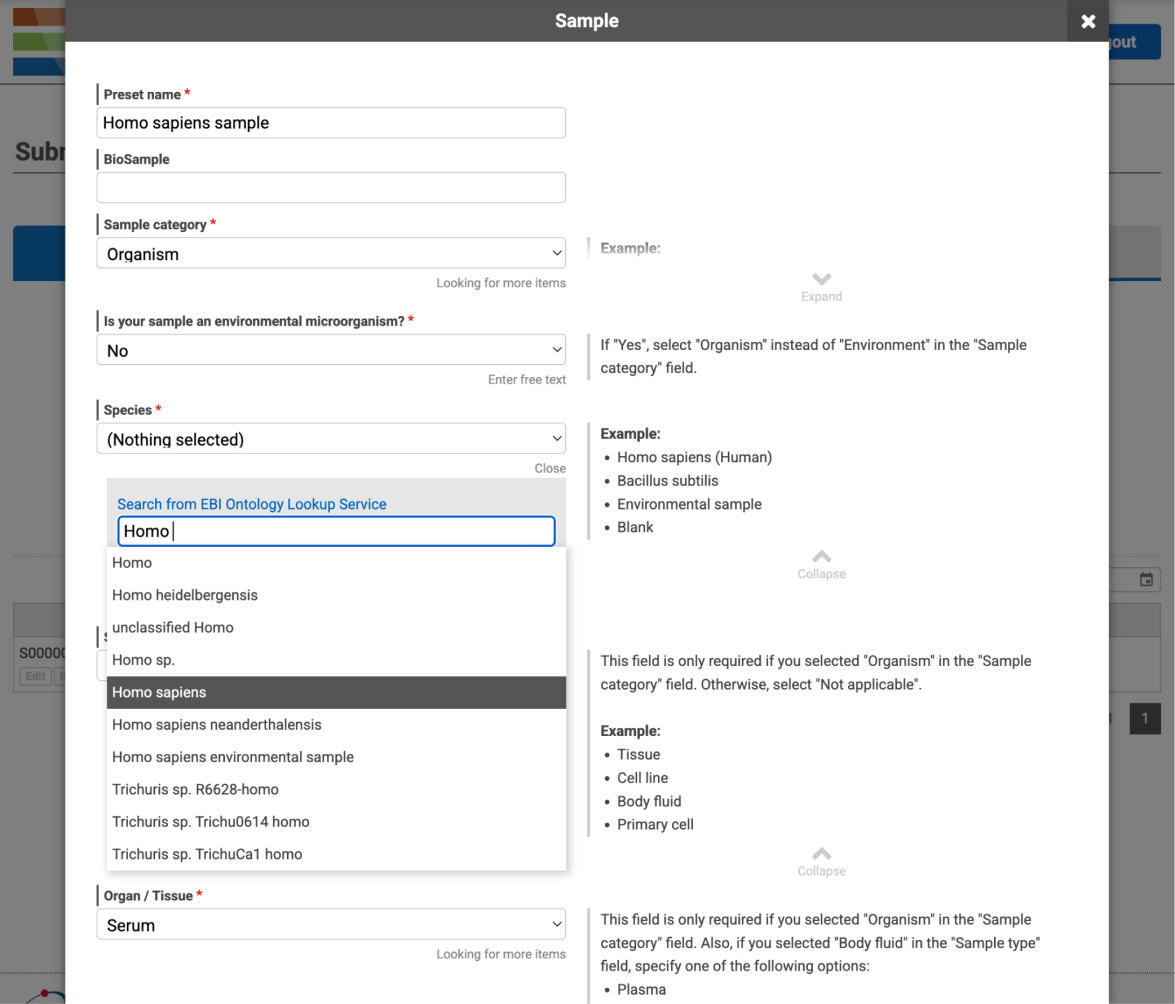
Screenshot of the “Sample” preset input interface in MB-POST. Incremental search enables users to select controlled terms beyond predefined options, while contextual explanations and examples for key metadata fields are displayed in the right panel.

All the metadata entered through the interface are automatically stored alongside their corresponding ontology identifiers to ensure full machine readability. Consequently, the use of ontologies and controlled vocabularies (CVs) is enforced transparently, ensuring consistent machine-readable metadata without increasing the cognitive or operational burden on users. This ontology-based controlled vocabulary framework, validated in jPOST, was optimized for metabolomics mass spectrometry data in MB-POST, enabling both rigorous semantic standardization and high usability to support reliable data reuse and large-scale integrative analyses. We have successfully mapped the majority of metadata to ontology identifiers by utilizing a variety of ontologies and CVs currently available; however, for terms for which ontology identifiers are not defined—such as ion chromatography–mass spectrometry (IC–MS) and supercritical fluid chromatography–mass spectrometry (SFC–MS)—we have associated them with identifiers from our custom-defined controlled vocabulary. Furthermore, MB-POST makes metadata seamlessly integrable with other resources as linked open data (LOD) by preparing metadata representations in the resource description framework (RDF), which is increasingly important for large-scale data integration and future applications, including those involving large language models (LLMs). Table S2 shows all metadata items included in each of the four preset categories, along with the corresponding ontologies and CVs for each item.

## Results and Discussion

Submitting experimental data to MB-POST requires users to first create a “project”, serving as a container for the data set. Each project is assigned a unique accession number by MB-POST, enabling all data sets reported in a single publication to be deposited in a single project. Alternatively, when data sets originate from multiple independent experiments, users may choose to divide them into two or more projects. A screenshot of the project-creation interface is presented in Figure S3.

After the project is created, the users select the files to be included. Each project must contain at least one raw data file, one identification-result file, and one replicate file. While MB-POST accepts raw data files in any format, we recommend submitting them in a standard format such as mzML^28^ whenever possible to maximize the reusability of the data. In addition, it is recommended that processed data matrices be submitted in the mzTab-M format^29^ along with annotation results as identification result files. These minimum requirements largely align with the partial submission criteria defined by the ProteomeXchange Consortium in the field of proteomics. For each raw data file, at least one preset must be assigned from each of the following four metadata categories: sample, preparation, analytical conditions, and software settings. Accordingly, users are required to prepare the presets for each project in advance. Presets may be created before or after the project creation.

Users can filter the preset list using keywords or the last update date when selecting the presets. Once the desired preset has been selected, multiple raw data files can be assigned the same preset profile simultaneously by selecting them together in the file selection dialog box or by dragging and dropping them onto the web interface. A screenshot of the preset assignment interface is shown in Figure 2.

**Figure 2:**
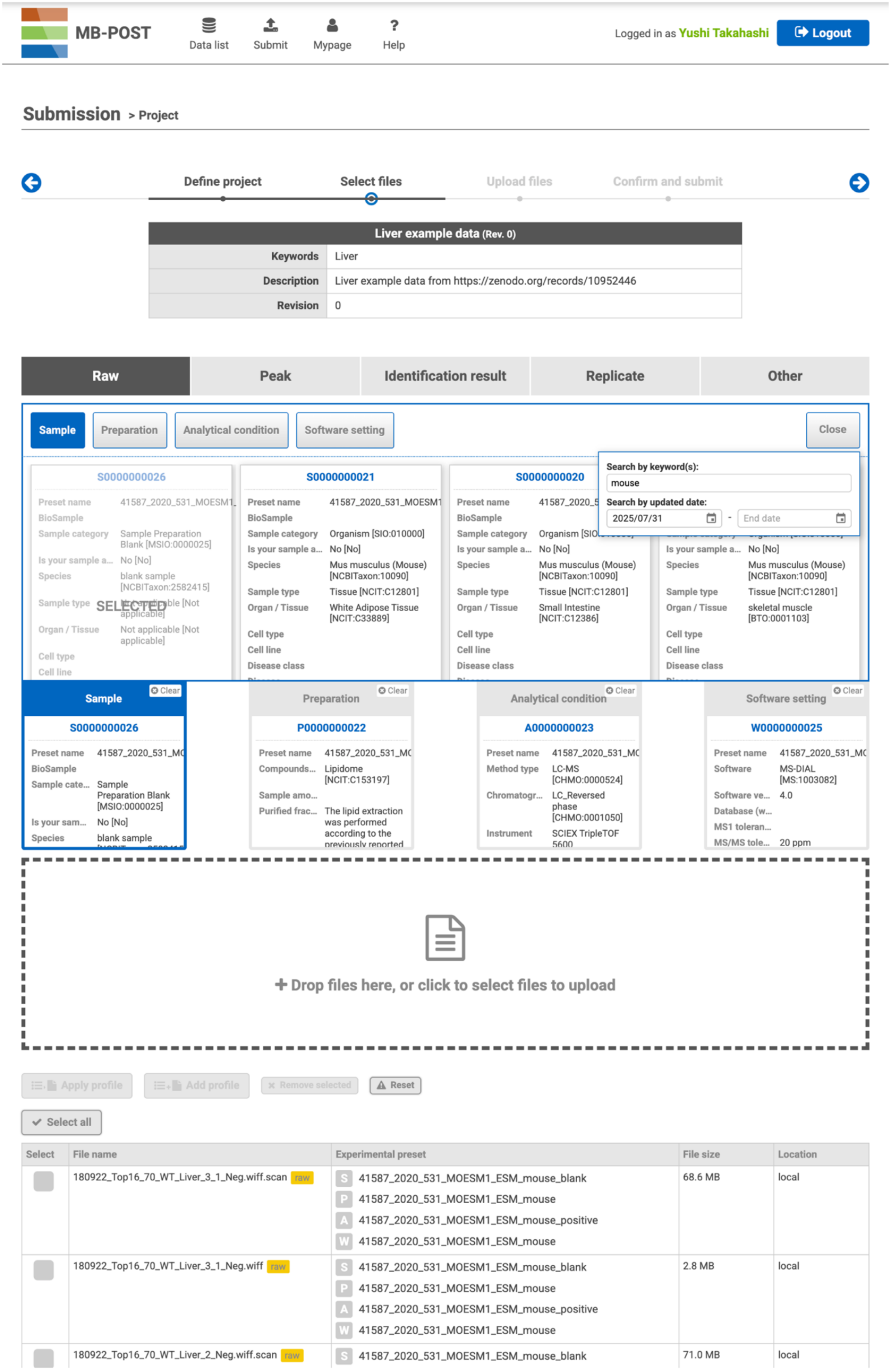
Screenshot of the interface for assigning preset profiles to files within a project. Users select the file type to be uploaded (“Raw”, “Peak”, “Identification result”, “Replicate”, or “Other”) using the tabs at the top of the screen. For raw data files, users first select the desired combination of presets from the preset list displayed immediately below, then drag and drop the target files into the dotted area on the screen. This operation enables the same preset profile to be assigned to multiple target files simultaneously. A table at the bottom of the screen displays a list of files associated with the current project, together with the names of the presets already assigned to each file.

For replicate files, MB-POST requires an Excel spreadsheet in a specific format that describes three types of replicate information for each raw data file: biological replicates, technical replicates, and injection replicates. Biological replicates follow conventional definitions used in experimental biology. When samples are processed without subdivision into multiple aliquots until injection into the GC–MS or LC–MS system, the resulting measurements are classified as injection replicates. In contrast, when the samples are divided into multiple containers prior to injection, and identical sample preparation procedures are independently performed for each container, these measurements are classified as technical replicates.

After the presets are assigned to each raw data file, users can download an Excel spreadsheet template via a web interface to enter the corresponding replicate information. This template file already contains the raw file names and assigned “Sample” preset names, enabling users to easily enter replicate information by simply filling in the three types of replicates highlighted in the red boxes. An example of a completed spreadsheet is shown in Figure S4.

After all raw data files have been annotated with preset profiles, and auxiliary files (e.g., peak lists, identification results, and replicate data) have been registered, users upload the complete set of project files to the MB-POST server. Figure 3 illustrates the relationship between the deposited data sets (projects) and metadata (presets) in MB-POST.

**Figure 3:**
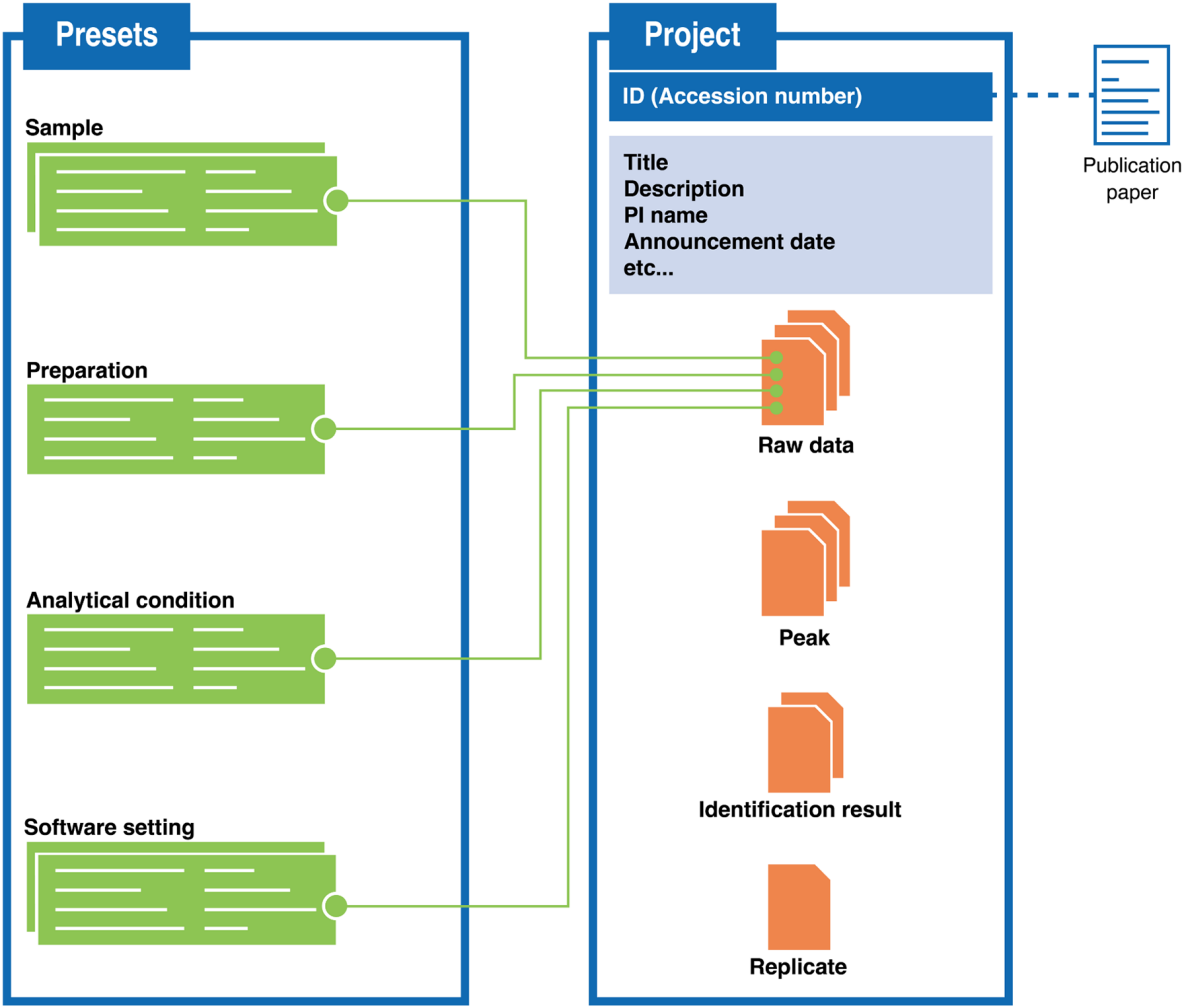
Conceptual overview of metadata assignment in MB-POST. Users create a single “project” that contains a submitted data set. Each project may include multiple files, such as raw data, peak list data, identification results, and replicate data. A combination of presets from four metadata categories—Sample, Preparation, Analytical Condition, and Software Setting—is assigned for each raw data file.

MB-POST supports fast and reliable file uploads using the PRESTO system, achieving transfer speeds of over 5 megabytes per second independent of location (within ∼4 min for 1 gigabyte files). Once all data files have been uploaded and the system confirms that the project meets all the submission requirements, users can lock the project to complete the submission. Upon locking, a unique accession number is assigned to the project. However, this does not imply an immediate public announcement.

Importantly, MB-POST provides features that directly reflect the needs of researchers in preparing manuscripts for publication. These include project-level embargo control, the generation of secure reviewer-only uniform resource locators (URLs) for peer review, and a revision function that enables submitted data sets to be updated in response to reviewer comments prior to public release.

In MB-POST, submitted projects are placed under an embargo by default unless the user explicitly requests immediate public disclosure. An embargoed project remains nonpublic until it is automatically announced by the system on the project announcement date specified by the user. If a user prepares a manuscript describing the experiments that generated the data set deposited during the embargo period, a dedicated URL and a personal identification number (PIN) code can be issued via the MyPage interface (https://repository.massbank.jp/mypage), enabling exclusive access to journal editors and peer reviewers. Furthermore, if a peer review results in requests for modifications to the deposited data set, such as adding files or supplementing metadata, the user can revise the project directly through the same MyPage interface. Initiating a revision unlocks the project, thereby enabling the user to add or replace experimental data files and modify the metadata as needed. Each revised project is assigned a revision number to ensure traceability. A flowchart illustrating the complete workflow, from preset and project registration through peer review and revision to the eventual public data announcement, is shown in Figure 4.

**Figure 4:**
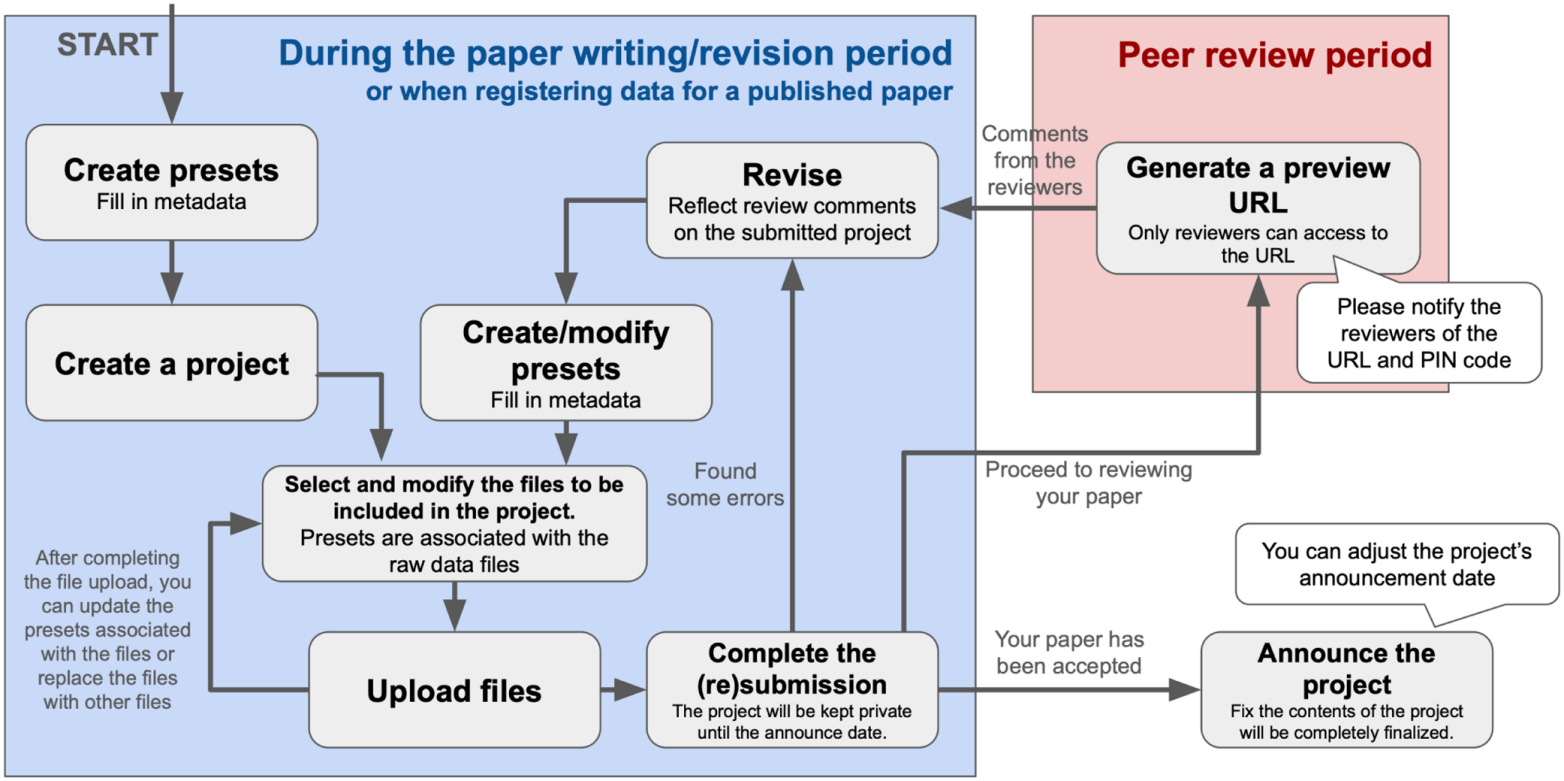
Workflow from preset and project registration to public release of the deposited data. Users can generate a preview URL accessible only to reviewers, together with a PIN code, by depositing the experimental data in MB-POST prior to manuscript submission, and provide this information during peer review. Because MB-POST fully supports embargoed submissions, users can independently revise deposited projects without assistance from system administrators in response to reviewer requests during the review process, provided that the project has not yet been made publicly available.

After the embargo expires, the projects are automatically released for public access. Public data sets can be freely browsed and downloaded by researchers worldwide without restrictions. Released projects are listed on the MB-POST Welcome page and can be searched using keywords derived from registered metadata, facilitating efficient data discovery and reuse. All data submitted to MB-POST are licensed under the Creative Commons CC0 1.0 Universal (CC0 1.0) Public Domain Dedication license. Detailed instructions and tutorial videos for MB-POST are available on the Help page (https://repository.massbank.jp/help), which users can access at any time from the navigation menu that is always displayed at the top of the screen. The source code for all MB-POST systems, except the PRESTO system, is kept closed-source. This is a deliberate decision designed to allow us to make our own decisions regarding development policies, release cycles, API changes, and so on.

## Conclusions

As described above, MB-POST also serves as a repository for newly acquired MassBank data and is publicly available as a subdomain on the MassBank website (https://repository.massbank.jp). The beta version was released in December 2024, followed by data deposition and system debugging by the development team and affiliated users. The official release took place in October 2025, after which submissions from general users, primarily from within Japan, began.

As of May 2026, 170 projects comprising 2.7 terabytes of data have been deposited in MB-POST within a year and a half, representing an unusually rapid uptake for a newly established metabolomics repository. Notably, more than 50 projects were submitted by general users within approximately two months, indicating that data deposition can be achieved with minimal effort. Currently, 66 projects are publicly available, and the remaining data sets are under embargo or in the process of being edited.

The defining features of MB-POST can be described as follows:

(i) Reanalysis-oriented design philosophy with sufficient metadata for MassBank integration and reproducible reanalysis.
(ii) Adoption of the proven jPOST infrastructure, providing high-speed uploads and user-friendly interfaces, has been validated over the past decade.
(iii) Comprehensive machine-readable metadata management using ontologies and controlled vocabularies: The ontology and controlled vocabulary management environment of jPOST were carefully optimized and reimplemented to meet the specific requirements of metabolomics.

As a result of these design choices, MB-POST enables the accumulation of metabolomics mass spectrometry data with accurate, standardized, and reanalysis-ready metadata while imposing the minimum possible burden on data contributors.

Future developments will initially focus on improving support for mass spectrometry imaging data, whose use is rapidly expanding. While currently submittable by designating coordinate/image information as an “other” file, a dedicated interface would facilitate deposition. Additionally, nuclear magnetic resonance data will be supported through metadata updates and system expansion, leveraging the experience from the current mass spectrometry data site. We also plan to add programmatically accessible APIs and command-line support for uploading and downloading data for expert users in the future.

Compared with other omics disciplines, metabolomics still lacks fully established standards for data publication, despite covering some of the most broadly reusable chemical data. The recently established MetabolomicsHub consortium is expected to serve as a central platform for international data repository collaboration in metabolomics, much like the ProteomeXchange consortium serves for proteomics. To maximize the long-term sustainability and data accessibility of MB-POST, we intend to join this consortium and update our system as necessary to comply with the guidelines established there. Under the principles of open science, MB-POST aims to maximize the reuse of metabolomics data across the chemical sciences and beyond while continuing to promote the interoperability and integration among repositories.

## Supplementary Material





## Notes

Conceptual diagram of file uploading in MB-POST using the PRESTO system (Figure S1); screenshot of the preset list interface of the MB-POST system (Figure S2); screenshot of the project creation interface (Figure S3); Excel spreadsheet for entering replicate information (Figure S4) (PDF)
The number of metadata items in existing metabolomics data repositories and MB-POST (Table S1); list of metadata items included in each of the four MB-POST preset categories and their corresponding ontologies and controlled vocabularies (Table S2) (XLSX)
