# Supplementary material for: Reviving Data Potential—The Reanalysis-Driven Metabolomics Data Repository MB-POST
Source: Anal Chem. 2026 Jul 27;98(31):22609–17. doi: 10.1021/acs.analchem.6c00697 (PMC13470969; doi:10.1021/acs.analchem.6c00697)
Supplement: Supplementary file 1 [file ac6c00697_si_001.pdf]

Supporting Information for

Reviving Data Potential – the Reanalysis-Driven Metabolomics Data Repository MB-POST

Yushi Takahashi[1†], Akiyasu C. Yoshizawa[1†], Takato Kiuchi[1], Ryosuke Hayasaka[2], Taihei Torigoe[3], Masatomo Takahashi[3], Takaki Oka[4], Yuki Matsuzawa[5], Motohiro Ogawa[4], Yoshihiro Izumi[3], Hiroshi Tsugawa[4,5], Akiyoshi Hirayama[2], Fumio Matsuda[6*], and Shujiro Okuda[1*]

†) Equal contribution
*) Co-corresponding authors

1. Medical AI Center, Niigata University School of Medicine, 2-5274, Gakkocho-dori, Chuo-ku, Niigata, 951-8514, Japan
2. Institute for Advanced Biosciences, Keio University, Kakuganji 246-2, Mizukami, Tsuruoka-shi, Yamagata 997-0052, Japan
3. Mass Spectrometry Center, Graduate School of Science, The University of Osaka, 1-1 Machikaneyama, Toyonaka, Osaka 560-0043, Japan
4. Department of Biotechnology and Life Science, Tokyo University of Agriculture and Technology, 2-24-16 Naka-cho, Koganei-shi, Tokyo 184-8588, Japan
5. Graduate School of Pharmaceutical Sciences, Kyoto University, 46-29 Yoshida-Shimoadachi-cho, Sakyo-ku, Kyoto 606-8501, Japan
6. Department of Bioinformatic Engineering, Graduate School of Information Science and Technology, The University of Osaka, 1-5, Yamadaoka, Suita, Osaka 565-0871, Japan

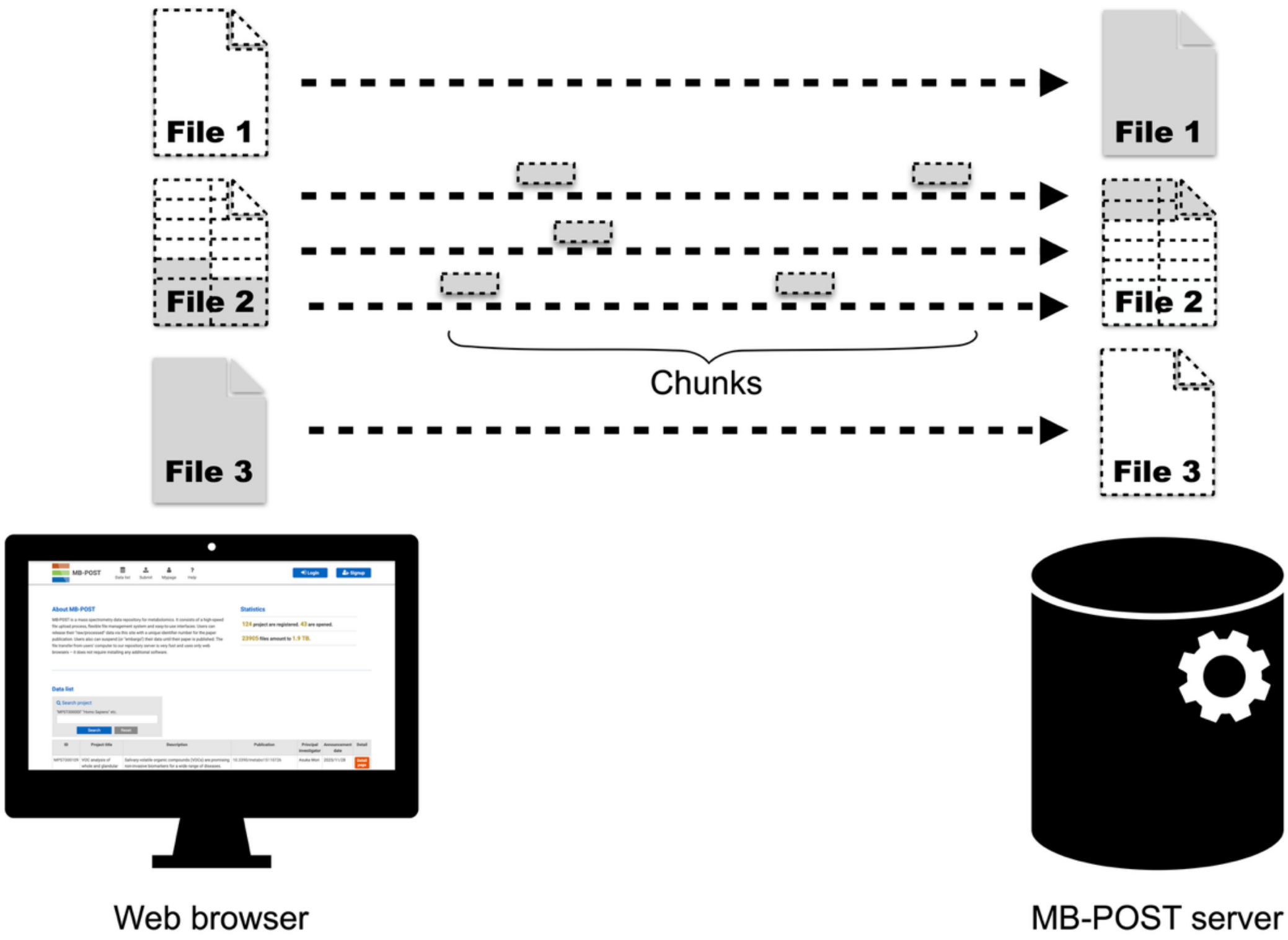

Figure S1. Conceptual diagram of file uploading in MB-POST using the PRESTO system. Each data file is divided into small chunks and uploaded to the MB-POST server in parallel. After all chunks have been successfully transferred, they are merged on the server side to reconstruct the original file.

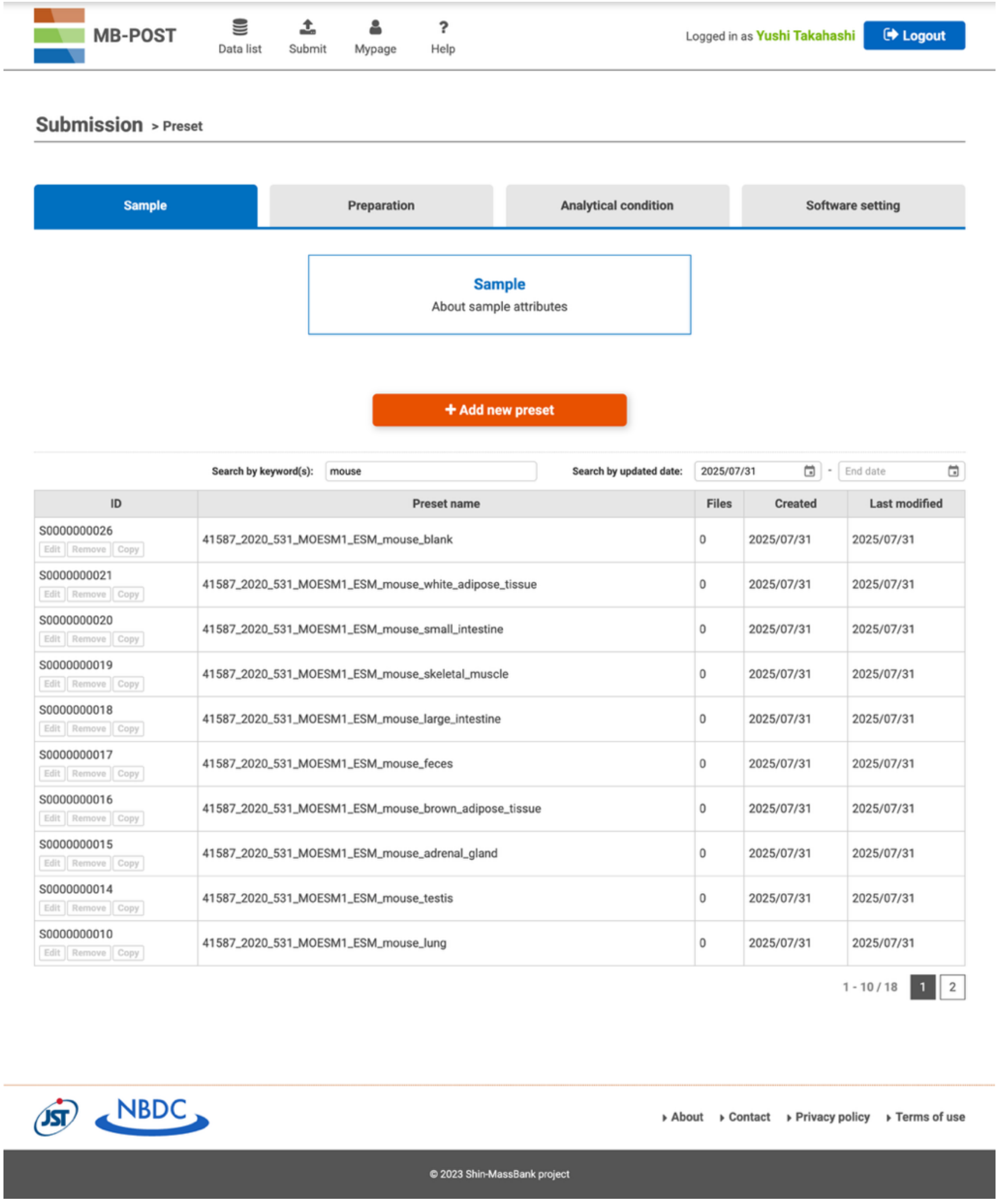

Figure S2. Screenshot of the preset list interface of the MB-POST system (https://repository.massbank.jp/preset). Presets are displayed in a tabular format and organized into four predefined preset categories; the displayed category can be switched by clicking the corresponding tabs at the top of the screen. Presets that are not yet associated with any file can be modified by clicking the "Edit" button, or deleted by clicking the "Remove" button, both located in the leftmost "ID" column. In addition, new presets with closely related settings can be efficiently generated by clicking the "Copy" button to duplicate an existing preset as a template. The text fields above the table allow users to filter the displayed presets based on keywords or the date of last update.

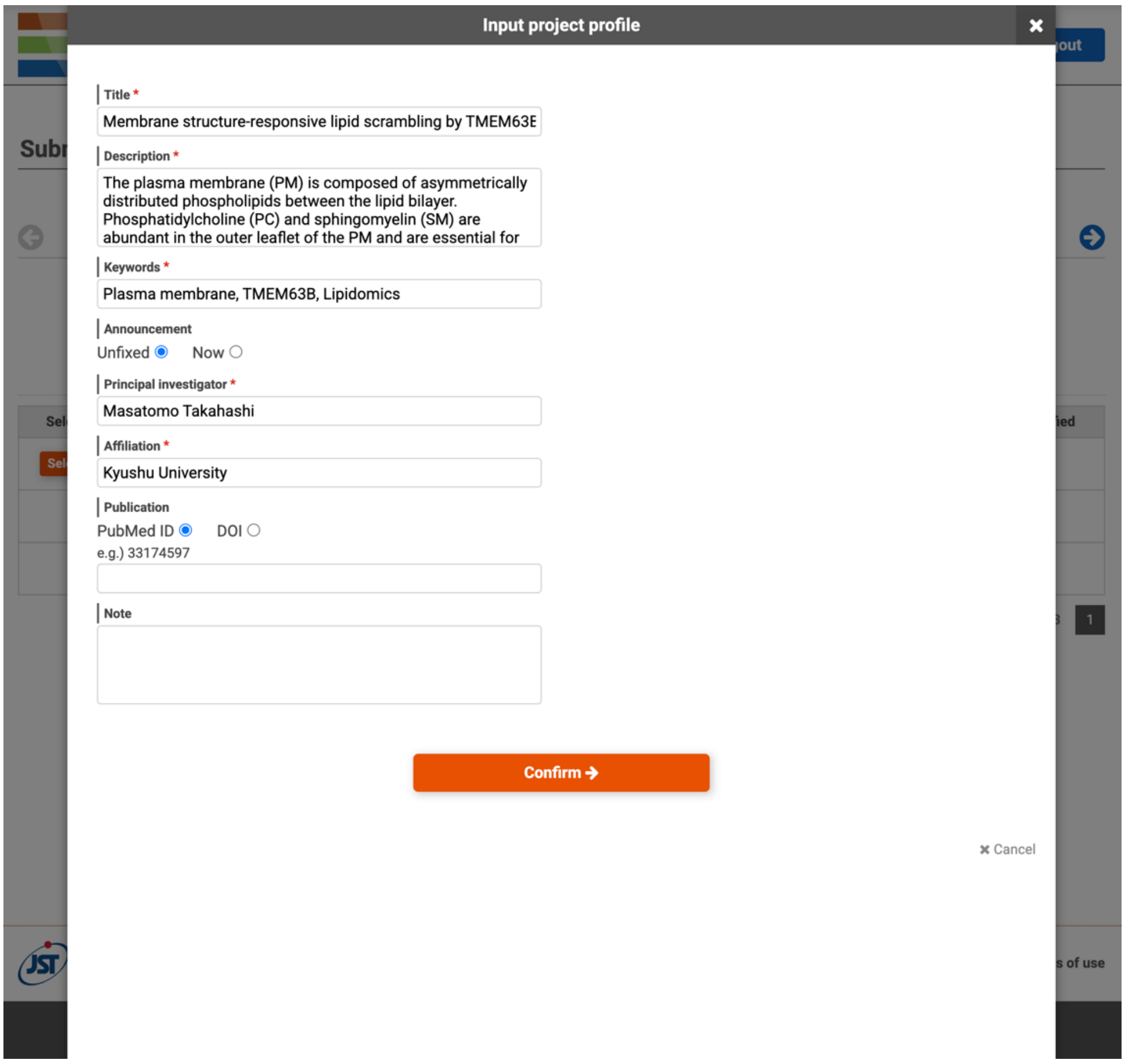

Figure S3. Screenshot of the project creation interface. Each project must include a title, description, keywords, and the name and affiliation of the principal investigator. If "Now" is selected as the 'Announcement' field, the project is made publicly available immediately upon completion of submission. If "Unfixed" is selected, the project remains under embargo and is not publicly accessible after submission. The provisional project announcement date is automatically set to one year after the submission date; however, users may freely modify it via the My Page screen (https://repository.massbank.jp/mypage).

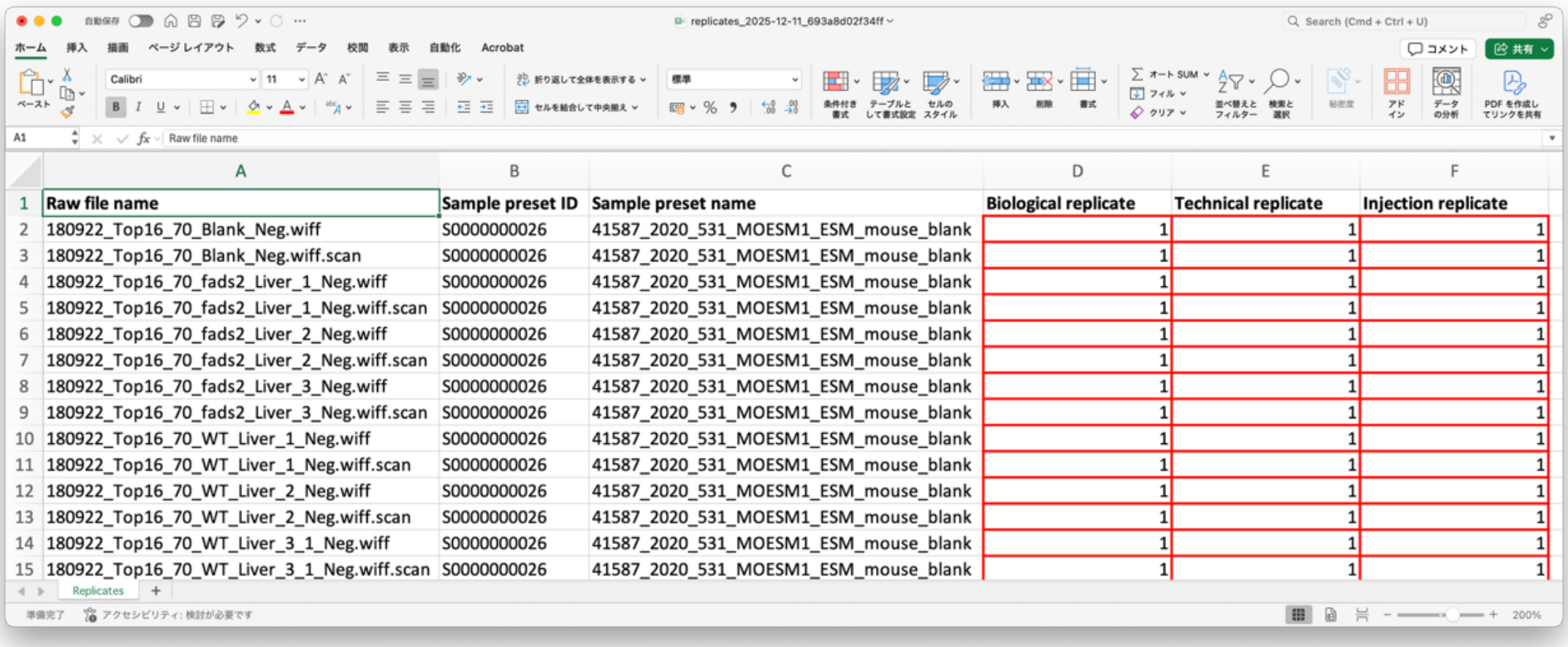

	A	B	C	D	E	F
1	Raw file name	Sample preset ID	Sample preset name	Biological replicate	Technical replicate	Injection replicate
2	180922_Top16_70_Blank_Neg.wiff	S0000000026	41587_2020_531_MOESM1_ESM_mouse_blank	1	1	1
3	180922_Top16_70_Blank_Neg.wiff.scan	S0000000026	41587_2020_531_MOESM1_ESM_mouse_blank	1	1	1
4	180922_Top16_70_fads2_Liver_1_Neg.wiff	S0000000026	41587_2020_531_MOESM1_ESM_mouse_blank	1	1	1
5	180922_Top16_70_fads2_Liver_1_Neg.wiff.scan	S0000000026	41587_2020_531_MOESM1_ESM_mouse_blank	1	1	1
6	180922_Top16_70_fads2_Liver_2_Neg.wiff	S0000000026	41587_2020_531_MOESM1_ESM_mouse_blank	1	1	1
7	180922_Top16_70_fads2_Liver_2_Neg.wiff.scan	S0000000026	41587_2020_531_MOESM1_ESM_mouse_blank	1	1	1
8	180922_Top16_70_fads2_Liver_3_Neg.wiff	S0000000026	41587_2020_531_MOESM1_ESM_mouse_blank	1	1	1
9	180922_Top16_70_fads2_Liver_3_Neg.wiff.scan	S0000000026	41587_2020_531_MOESM1_ESM_mouse_blank	1	1	1
10	180922_Top16_70_WT_Liver_1_Neg.wiff	S0000000026	41587_2020_531_MOESM1_ESM_mouse_blank	1	1	1
11	180922_Top16_70_WT_Liver_1_Neg.wiff.scan	S0000000026	41587_2020_531_MOESM1_ESM_mouse_blank	1	1	1
12	180922_Top16_70_WT_Liver_2_Neg.wiff	S0000000026	41587_2020_531_MOESM1_ESM_mouse_blank	1	1	1
13	180922_Top16_70_WT_Liver_2_Neg.wiff.scan	S0000000026	41587_2020_531_MOESM1_ESM_mouse_blank	1	1	1
14	180922_Top16_70_WT_Liver_3_1_Neg.wiff	S0000000026	41587_2020_531_MOESM1_ESM_mouse_blank	1	1	1
15	180922_Top16_70_WT_Liver_3_1_Neg.wiff.scan	S0000000026	41587_2020_531_MOESM1_ESM_mouse_blank	1	1	1

Figure S4. Excel spreadsheet for entering replicate information. Users are expected to enter replicate identifiers as integer values in the columns labeled "Biological replicate," "Technical replicate," and "Injection replicate," which are highlighted by red boxes. All other fields in the spreadsheet are prepopulated by the system and should not be modified.
